# 非气管插管在胸外科VATS中的应用

**DOI:** 10.3779/j.issn.1009-3419.2016.05.09

**Published:** 2016-05-20

**Authors:** 小探 代, 平平 宋, 百江 张

**Affiliations:** 1 250117 济南，济南大学医学与生命科学院 School of Medicine and Life Sclences, University of Jinan, Jinan 250117, China; 2 250117 济南，山东省医学科学院 Shandong Academy of Medical Sciences, Jinan 250117, China

**Keywords:** 电视胸腔镜手术, 非气管插管, 微创, Video-assisted thoracic surgery, Non-intubated anesthesia, Minimally Invasive

## Abstract

气管插管全麻技术可提高手术安全性，因此在电视胸腔镜手术（video-assisted thoracic surgery, VATS）中得到广泛应用，但气管插管的并发症却无法避免。如何发展一种“整体微创”手术（包括麻醉微损伤），已经成为微创胸外科领域的一个研究热点。随着麻醉管理技术与对手术风险管理的进步，非气管插管技术成功应用于VATS，即采用局部麻醉以维持患者的术中自主通气，术中仅需轻微镇静或者完全清醒的状态下实施VATS，因而又称清醒状态下VATS。此麻醉方式不但减少气管插管的麻醉损伤，而且符合快速康复外科的理念。本文对非气管插管应用在胸外科VATS中的发展简史、麻醉选择、手术优势、手术风险及管理、面临的问题等方面作一综述。

## Abstract (en)

Tracheal intubation general anesthesia technique is widely used in video-assisted thoracic surgery (VATS) because it can improve the safety of VATS, but the complications of tracheal intubation can not be avoided. How to develop a "minimally invasive" surgery (including micro anesthesia) has become a hot topic in the field of minimally invasive surgery. Along with the progress of the anesthesia management technology and the risk management in the operation, the technology of non-intubated anesthesia was successfully applied to VATS, namely using local anesthesia to maintain patients intraoperative independent ventilation and intraoperative only mild sedation or fully conscious state of implementation of thoracoscope surgery, therefore is also called awake VATS. The anesthesia method not only reduces the anesthesia injury of tracheal intubation, but also conforms to the idea of rapid rehabilitation surgery. Based on non-intubated anesthesia in VATS in the brief history of development, the anesthesia selection, operation advantages and risks are reviewed in this paper.

## 1 非气管插管应用在VATS中的发展简史

这项技术的关键是麻醉技术与对手术风险管理的不断进步。自2004年Pompeo教授首次报道^[1]^非气管插管技术成功应用在电视胸腔镜手术（video-assisted thoracic surgery, VATS）中至今，国内外关于这方面的研究一直没有停止。随着麻醉技术的进步，早期报道单纯行胸段硬膜外麻醉可增加支气管张力及气道反应性，术中牵拉肺组织激发咳嗽反射，干扰手术操作及进程^[1]^，对此采取术前半小时雾化吸入利多卡因^[2]^、术中局部浸润麻醉星状神经节^[3]^、迷走神经阻滞^[4]^等措施，这些措施抑制咳嗽反射的效果令人满意；另外，早期术中对患者镇静深度只能凭借麻醉医生的主观经验，到现在实施麻醉的同时常规监测患者脑电双频指数（bispectral index, BIS）^[5]^，为精准麻醉提供了客观指标；最后，由早期单一的单纯行硬膜外麻醉发展到现在的量身定制组合各种麻醉模式。随着手术风险管理的进步，报道的重点从早期处理胸腔积液、自发性气胸、肺大泡切除术、肺结节切除术等简单的胸部疾病，到现在复杂的肺癌根治术、胸腺切除术等。可以看出麻醉技术与对手术风险管理的不断进步不仅使非气管插管技术应用于VATS成为可能，同时主导着清醒状态下VATS走向成熟。

## 2 非气管插管VATS的麻醉选择

传统VATS麻醉方式为全麻。但全麻并发症较多。而且，无法避免因气管插管单肺通气产生的非通气侧肺损伤，缺血再灌注损伤^[6]^。而非气管插管局麻技术可保留患者术中的自主呼吸，维持正常呼吸状态，可避免因气管插管带来的并发症。为不能耐受气管插管全麻的老年患者增加了一种新的麻醉选择^[7]^。不但如此，现在的量身定制组合各种麻醉模式，包括静脉麻醉、术中迷走神经阻滞、胸膜表面浸润麻醉、肋间神经阻滞、椎旁神经阻滞及硬膜外麻醉等^[8-11]^，可根据患者的具体情况和术式而定。明显减少了全麻手术对患者应激激素的释放^[12]^及免疫功能的损伤^[13]^。其中硬膜外麻醉应用最为广泛^[4]^。通气方式可以选择喉罩、面罩、鼻咽通气道等^[14]^。

### 2.1 肋间神经麻醉+静脉镇痛镇静^[15]^

局部浸润麻醉手术切口所在肋间的肋间神经，同样可以满足术中操作所需的镇痛强度^[3]^。台湾学者陈晋兴教授用2%利多卡因局部浸润切口部位，待人工气胸形成后，在胸腔镜直视下用0.5%布比卡因浸润胸膜壁层第三到第八肋交感神经链外侧2 cm处（每个肋间1.5 mL）。鉴于早期术中对患者镇静深度只能凭借麻醉师的主观经验，并无客观指标，因此，现在实施麻醉的同时监测BIS^[5]^。术中采用靶浓度控制输注模式（target controlled infusion, TCI）输注异丙酚静脉进行镇静镇痛治疗。将BIS值维持在40-60之间，后追加静脉注射芬太尼25 mg，保持呼吸频率维持12次/min-20次/min之间以便于手术操作。

### 2.2 超声引导下胸椎旁神经阻滞^[16]^

意大利医生Piccioni首次报道在VATS中使用此麻醉方式处理胸腔积液。他选择将T3-T6棘突旁作为穿刺点，用7 mL 1%利多卡因浸润皮肤皮下后进针，给药方式为经椎旁间隙导管注射5 mL 1%罗哌卡因每段。研究报道术中血流动力学稳定，耐受性气胸良好，无咳嗽。并指出麻醉方式优点在于仅阻滞手术侧躯体，机体生理影响小，而不足之处为气胸发生率5%。

### 2.3 胸段硬膜外麻醉+静脉镇痛镇静+迷走神经阻滞^[4]^

此麻醉方式在非气管插管VATS中应用最为广泛，其中广医附院何建行教授选择在T8-T9行硬膜外穿刺，进针后先给予2 mL试验剂量的2%利多卡因，无异常后再注入罗哌卡因，并根据术中手术部位、手术操作及进程对镇静或镇痛的需求不同，调整镇静或镇痛程度。常用的丙泊酚和瑞芬太尼效应室靶浓度分别为0.5 μg/mL-1.5 μg/mL和0.5 ng/mL-2.0 ng/mL。而迷走神经阻滞则由手术医生在术中实施，经术野用2 mL-3 mL的2%利多卡因局部注射迷走神经干旁粘膜下（注意两侧迷走神经解剖有所不同）。

### 2.4 麻醉排除标准

虽然已有大量研究报道非气管插管技术的可行性，但保证患者术中安全依然最重要，因此做好术前麻醉排除很重要。非气管插管VATS实质为非气管插管麻醉结合VATS。然而非气管插管气道维持安全性差，因此术前需严格筛选患者，另外根据GonzalezRivas团队及其他主要研究小组的经验^[17]^，需要对患者进行充分严格的筛选，故有以下情况的患者，同样不适合应用非气管插管技术。①被麻醉医生评估为气道管理困难；②血流动力学不稳定；③肥胖[身体质量指数（body mass index, BMI）>30 kg/m^2^]；④凝血功能障碍（国际标准化比值>1.5）；⑤持续性咳嗽或气道粘液高分泌；⑥存在高返流风险；⑦神经系统疾病：癫痫发作的风险，无法配合，颅内或脑水肿；⑧广泛胸膜粘连或曾经肺切除（与外科医生的技术有关）；⑨低氧血症（PaO_2_ < 60 mmHg）或高碳酸血症（PaCO_2_>50 mmHg）；⑩中枢性低通气综合征；⑪须肺隔离技术，以保护对侧肺免受污染。

## 3 非气管插管应用在VATS中的优势

### 3.1 国外研究

早期国外关于VATS中应用非气管插管的优势由Pompeo教授^[1]^率先报道，他进行一项随机试验，比较60例周围型肺结节患者的手术结果，鉴于当时术中麻醉管理技术及术前相关辅助检查的限制，非气管插管组术中血氧饱和度显著低于气管插管组（3 mmHg *vs* 6.5 mmHg, *P*=0.02），且术中2例患者因胸膜严重粘连需要中转开胸。而在麻醉满意度（4分*vs* 3分，*P*=0.04）、术后护理（每天呼叫次数2.5次*vs* 4次，*P*=0.000, 1）及住院天数（2 d *vs* 3 d, *P*=0.02）方面较气管插管组显著优越。之后有研究^[18]^多角度报道了45例原发性多汗症的手术优势，研究显示，虽然两组患者在远期生活质量方面没有统计学差异，但非气管插管在麻醉药量、手术时间、住院天数及手术后24 h患者满意度与对照组对比有统计学意义。除此之外，多位学者的研究^[19-27]^均肯定了非气管插管麻醉下VATS的可行性及手术效果。然而，这些研究存在样本队列小，单一中心设计，而且各自研究都是由单一手术团队完成等弊端。因此，Jun及其团队^[14]^进行了目前样本量最大的一项研究，354例接受包括肺大疱切除术，肺楔形切除术和肺叶切除术的患者随机入组到硬膜外麻醉的实验组（*n*=174）和气管插管全麻的对照组（*n*=180）。非插管麻醉组术后可恢复进食时间（7.8 d *vs* 19.9 d, *P* < 0.001）、使用抗生素时间（2 d *vs* 4 d, *P* < 0.001）及住院天数（5.8 d *vs* 7.7 d, *P* < 0.001），与对照组相比存在统计学差异。除此之外，实验组行肺大疱切除术的患者术后的支气管肺泡灌洗液中的肿瘤坏死因子-α（tumor necrosis factor-α, TNF-α）浓度和高敏C反应蛋白（supersensitivity C reactive protein, hs-CRP）与对照组相比有统计学意义（*P*=0.038）。

### 3.2 国内研究

国内关于在VATS中应用非气管插管的优势最先由贺钢枫报道^[28]^，他对8例患者实施非气管插管VATS，术后诊断为结核病变，转移性癌，炎性假瘤及间质肺纤维化等肺部疾病。结果提示除一例因肺脓肿中转开胸外，其余7例均在自主呼吸下安全完成肿物切除。术中血压、脉搏及氧饱和度均在正常范围内，术后无围手术期并发症和死亡。报道指出此术式经济（费用为双腔插管全麻VATS肺楔形切除术的1/2）、微创、利于普及应用。越来越多的学者尝试应用这种新的麻醉方式，广医附院何建行教授报道非气管插管VATS楔形切除术202例，结果示术中生命体征平稳，无一例中转为全麻气管插管，进胸前后平均动脉压和SPO_2_无统计学差异[(71.1±12.2) kPa *vs* (99.1±2.7) kPa, *P*>0.05）]。虽然肺氧合指数会在开胸术后15 min明显下降至（289.5±141.7），但在30 min左右恢复到（410.5±117.6）。而且在关闭伤口后15 min后平均动脉压、呼吸频率和SPO_2_会恢复至麻醉前水平；术后60 min内pH与PaCO_2_恢复正常。证实非气管内插管在VATS肺楔形切除肺大疱肺结节是可行的^[4]^。国内多位学者的研究均报道了非气管插管技术在VATS中的应用价值^[29-34]^。随着麻醉管理技术与对术中风险管理的不断进步，非气管插管被尝试应用在复杂的肺切除术中^[35]^。何建行教授通过对15例非气管插管肺段切除术进行回顾性分析^[8]^，以评估这项技术的安全性和可行性，结果示手术顺利完成，术中无开胸，证实非气管插管是一种安全、可行的技术，可用于治疗原发性肺癌、肺转移和良性疾病。更多的学者同样进行这方面的研究，台湾学者陈晋兴教授对280例肺肿瘤和5例气胸患者实施非气管插管VATS治疗，术式包括137例肺叶切除术、16例肺段切除术和132例楔形切除术，结果示无一例死亡，术中仅14例中转为气管插管，1例中转为开胸，患者术后立即返回日常生活中，证实非气管插管VATS肺切除是安全可行的^[36]^。

## 4 VATS非气管插管的手术风险及管理

### 4.1 手术风险

众所周知，非气管插管气道维持安全性差，术中易出现胃内容物反流与误吸等风险，同时根据Jose Navarro-Martinez对非气管插管VATS的术中风险管理的研究^[37]^，其报道手术风险主要为中转为气管插管全麻VATS甚至传统开胸手术，原因如下：①呼吸性酸中毒，pH < 7.1；②缺氧（PaO_2_ < 60 mmHg）仍未改善；③尽管雾化吸入利多卡因和迷走神经阻滞，咳嗽仍无法改善；④焦虑发作与镇静无改善；⑤患者自愿转换；⑥大出血；⑦肺胸壁广泛粘连超过50%；⑧较大肿瘤，特别是在最中央和前肺。

### 4.2 风险管理

① 针对非气管插管气道维持安全性差，首先对患者进行严格筛选与术前准备，一旦术中出现胃内容物反流与误吸，须迅速中转气管插管全麻；②非气管插管VATS中允许性高碳酸血症很常见，通常具有很好的耐受性，无需特殊处理，以防万一，需准备一个非侵入的呼吸机；③严重低氧血症情况下，首先使用高流量氧通气，通常为20 L/min-30 L/min和100%的氧浓度。若患者仍然持续低氧血症，可以使用无创通气。如果患者不耐受高流量通气，则气管插管是唯一解决方案；④在手术开始和气胸完成时，即使氧饱和度没有下降，患者仍会感到呼吸困难。做好术前沟通下，患者耐受性会很好，若呼吸困难持续存在，则需使患者安静下来；⑤术中最常见问题之一是咳嗽，在各项措施均未达到有效抑制情况下，可采用静脉注射利多卡因增强效果；⑥针对术中出现大出血情况，Jose Navarro-Martine的做法是在手术对侧导入2个外周静脉导管，在大失血情况下，快速输液，用海绵棒压住伤口的同时中转开胸手术，可用超声、电凝、一期缝合、肺动脉夹闭和吻合血管等手段止血；⑦求助，安排一名急诊外科领域的术者和在手术开始时候配备两位麻醉医生，在危机时刻向这方面的专家求助。因此不仅需要术中麻醉医生和外科医生密切配合，更要求两者准确判断术中风险并及时做出选择。

## 5 面临的问题

### 5.1 争论点

目前仍有学者对此新技术持不同意见：①能否真正做到在不伤害原则前提下让患者获益，诸如技术的实用性、安全性与具体手术指征等长期结果仍需要经过前瞻性多中心临床试验的大样本进一步证实^[38]^；②在早期全麻技术并不完善条件下，为了对一些特殊患者实施非气管插管手术，会对患者进行严格筛选，增加肺功能锻炼等术前准备；③前期术中无TCI模式输注和监测患者BIS等客观指标、患者主观感觉较差等问题。因此得出的结论不足以得到全部学者的认可。

### 5.2 开展与推广

① 因非气管插管安全性不高，因此术中需时刻警惕患者术中各项变化而增加麻醉医生的工作负担；②不同地区VATS技术、规模参差不齐，医生接受到培训也大相径庭^[39]^；③在设备与人才配备不良的国内基层医院，虽然乐于开展此类手术以节约医疗成本，但是存在一定的安全隐患问题；④国家如何提供更广泛深入的医疗保障。上述种种问题仍有待解决，进而限制了新技术的开展和推广。即使目前仍存在种种存在争议的地方，也应该清楚意识到作为一种新的麻醉方式，非气管插管在VATS中是有前途的，可作为一些适应症患者新的麻醉选择。

## 6 小结

胸外科VATS的微创优势已深入人心，然而，微创是一个不断发展的概念，新与旧，先进与落后，都是相对的。VATS技术仅能体现切口微创，随着微创技术的不断发展，对当代胸外科微创理念提出了更高的要求，不仅要求手术切口的微创，而且需要麻醉减少对患者的各器官功能的损伤。将非气管插管技术应用于VATS手术中，更准确的选择适应症^[39]^，让不同学科的专业优势更完美地配合，更好地推动了各学科的发展，也将使胸外科微创手术从切口微创发展到整体微创，进一步减少对患者的创伤，更有利于患者的术后快速康复，使更多的患者更切实地获益。
